# Ground effect of an inverted double element wing diffuser on a sedan car

**DOI:** 10.1016/j.heliyon.2024.e29435

**Published:** 2024-04-13

**Authors:** Mustafa Sabeeh Abood, IhsanYahya Hussain

**Affiliations:** University of Baghdad, Mech. Engr. Dept., Baghdad, Iraq

**Keywords:** Ground effect, Double-element inverted wing, Nissan sunny car, Car diffuser, CFD, Drag reduction, Passive flow control

## Abstract

The diffuser is a critical component in sports cars, enhancing aerodynamics by increasing downforce and reducing drag. Previous studies have focused on its dependence on diffuser incidence, height, and base pressure. The design of the car, particularly the rear end shape and the rear wing's presence, affect base pressure and the diffuser's performance. Previous studies have investigated the effects of diffuser geometry on aerodynamic performance, but the current study is the first to examine the relationship between the diffuser and the rear tires. It also provides specific and quantitative results on the impact of different diffuser design parameters on drag and downforce. The relationship between the rear tires and the double-element inverted wing diffuser using computational fluid dynamics (CFD) was investigated. This is an essential problem because the diffuser is a critical component of sports cars, and its design can significantly impact aerodynamic performance. CFD was used to simulate the flow of air around the car model. The CFD model was based on the Nissan Sunny (Versa) type Almera design, and the diffuser main element and flap wing angles were set at 4 and 15.5°, respectively. The flap gap, overlap distance, and wing ride height above the ground were varied to achieve an optimal aerodynamic design. The study found that the wing's ride height significantly influences the flow through the diffuser. The diffuser significantly impacts base pressure and downforce production. Increasing the ride height decreases base pressure, leading to an increase in downforce until a specific point near the car body, where downforce further increases. The study concluded that the best double-element diffuser design was selected based on lift-to-drag results and the allowable dimensions of the car, wing ride height, element gap, and overlap distances. Ultimately, the best diffuser wing design features a ride height of 154 mm, a gap distance of 10 mm, and an overlap of 5 mm. This design reduces drag by approximately 2.7 % and remarkably increases downforce ten times compared to the baseline car model.

## 1 Introduction

Effective aerodynamics is one of the most essential characteristics of a good modern car. Low aerodynamic drag is crucial for vehicles, much as for cars and trucks, as it decreases fuel consumption, which is an essential factor. Furthermore, a higher maximum speed is achieved by minimal aerodynamic drag. Negative lift, or downforce, is the most crucial element in car designs. More significant lateral acceleration may be attained through the turns of a particular racing course because of increased downforce, which increases the traction force of the tires.

The modern and racing car's ground-effect diffuser is often found near the rear of the underbody. The ground-effect diffuser displays an asymmetrical shape, typically comprising a single ramp surface that diverges, unlike the diffuser with a planar wall and symmetrically divergent sides. As the diffuser approaches the road surface, its cross-sectional area gradually expands, transitioning into an increasing area duct. It creates a space where the underbody airflow that enters the diffuser at high velocity and low pressure may depart at low velocity and high pressure [1]. Producing the maximum negative lift with the least drag penalty improves Formula 1 racing vehicle's aerodynamic performance as a significant automotive aerodynamic component [2]. The ground effect diffuser generates negative lift due to the effect of negative pressure formed below the racing car. The high-velocity airflow of the car's underbody floor goes beneath the smooth underbody floor. It travels through the diffuser region, which begins at the floor downstream and flows gradually, slowing to the low-velocity airflow at the diffuser's outlet [3]. Therefore, the diffuser's expanding surface makes it easier for low pressure airflow at its entrance to transition into higher pressure airflow at its outlet. As the airflow velocity decreases and pressure increases downstream of the inlet, a process known as pressure recovery occurs [4]. This ground effect phenomenon is what causes downforce to occur.

Diffusers have been an essential aerodynamic component of race cars for many years, and they have been shown to enhance downforce while reducing aerodynamic drag when the design is optimized. Cooper et al. [4] have shown that small, shallow-angle diffusers, as found in passenger cars, can achieve useful levels of drag reduction. A smooth, low-loss underbody flow channel would improve drag reduction in this application. Diffusers have been studied extensively, with a highlight on flat diffusers [5]. Three-dimensional ground effect diffusers have been analyzed for flow characteristics, drag, and lift [4]. The primary function of a diffuser is pressure recovery, but in automotive applications, it also interacts with the ground, pumping phenomenon, and underbody upsweep.

Cooper et al. [4,5] researched the phenomena of diffuser pumping. This effect arises from the vehicle's base pressure fixing the pressure at the diffuser outlet. This fixed outlet pressure reduces pressure at the diffuser inlet, which is believed to be the driving force behind the lowered underbody pressures as pressure increases along the diffuser's length. The resulting low pressure at the diffuser inlet is a significant source of downforce and increases the flow rate beneath the vehicle. The sweep angle on the diffuser on the diffuser will influence downforce production on the car in the same manner that an inverted wing would.

George [6] observed the development of a longitudinal vortex pair along the longitudinal sides of the diffuser. Due to an increase in the pitch of the bluff body, which happens when the nose of the body is down, the diffuser's effective incidence was raised, and the induced inflow caused the vortices to expand and strengthen. This expansion and enhancement of vortices prevented the formation of a separation bubble on the diffuser ramp. Skirts were attached to the sides of 10° and 15° diffusers to achieve this effect; George and Donis [7] observed that the diffuser flow was being stopped by a blockage in the input from the longitudinal-edge vortex pair. By gradually lowering the riding height from high to low, Senior (2000) and Senior and Zhang [8,9] identified four unique force regimes with comparable diffuser flow characteristics. Jowsey's research [10] shows that longitudinal fences that divide the diffuser flow channel produce smaller longitudinal vortices that increase downforce and improve diffus er sending out and pressure recovery. He conducted examinations on the underbody and the bluff body diffuser. At the same time, Puglisevich [11] carried out computational fluid dynamics (CFD) experiments using large eddy simulation (LES) to predict the surface pressure distribution geometry precisely. Marklund [12] noticed the diffuser performed at its most effective when the near wake symmetry was maintained, as observed in both experimental and CFD tests, depending on the Realizable turbulence model in the numerical test conducted on passenger automobiles and bluff bodies. Christoffersen et al. [13] used CFD techniques to investigate the interaction of a modern sports car-style race vehicle's diffuser and rear wheels. The fully detailed Lotus Evora Type 124 CUP automobile served as the geometry for the project. His investigation adjusted the diffuser angle to various inclinations, from 3 to 12°. Additionally, there were variations in the wing height above the "back deck" of the vehicle. It was observed that the wing's vertical position had minimal influence on the diffuser's flow at the examined heights. It was also observed that the diffuser had a negligibly small impact on the flow through the rear deck.

Cederlund and Vikström [14] have found that the complex shapes of modern sports vehicles significantly impact diffuser performance, base pressure, and other design factors. Rear-end components, such as the rear tires and rear wing, directly relate to diffuser performance and base pressure. The wake structures created by the rear tires can significantly impact the diffuser's airflow, pulling the diffuser's center inwards. This negatively impacts diffuser performance, so it is essential to consider the rear tires when designing a diffuser for a modern sports vehicle. The rear wing can also impact diffuser performance and base pressure, but racing regulations often limit its position. Sakran [15] addresses an essential aspect of vehicle aerodynamics and systematically investigates wake regions and drag coefficients. The study focused on explaining the results and correlating them with pressure and velocity distributions, enhancing the clarity and credibility of the findings. This research can benefit car manufacturers and designers seeking to optimize vehicle aerodynamics, ultimately leading to improved fuel efficiency and enhanced performance on the road. Ehirim et al. [16] conducted an experimental and computational investigation to explore a new approach to applying aerodynamic concepts to improve the ground effect diffuser of the automobile type's pressure recovery properties. Through validated CFD simulations compared to wind tunnel measurements, they observed an increase in negative lift along various ride heights tested, with the maximum increase reaching approximately 12 % compared to the baseline (without the wing). Faris [17] studied the aerodynamic characteristics of simple passenger car models. The study concentrated on analyzing the aerodynamic performance characteristics of a Peugeot car model and the effects of aerodynamic forces on drag and stability. Zahraa [18] has likewise carried out a number of studies on a simplified car model (KIA Pride) and the potential of reducing drag by adding a diffuser under the car, along with other modifications. In this study, the researchers utilized numerical modeling techniques and wind tunnel tests to analyze the airflow around the vehicle. They found that the implementation of an effective rear slice diffuser significantly improves the aerodynamic performance of the KIA Pride car model. As a result, it reduces drag forces and enhances stability during driving. These findings provide valuable insights for car manufacturers and designers, enabling them to optimize vehicle designs and increase their aerodynamic efficiency and on-road stability.

Tumse et al. [19,20] investigated the influence of ground proximity on the aerodynamic performance and flow properties of a non slender delta wing with a 40° sweep angle. The studies used particle image velocimetry (PIV), an aerodynamic force measurement system, and dye flow visualization. They analyzed the wing at various distances from the ground and at attacks of 8° and 11°. Their findings demonstrate that ground proximity significantly alters the behavior of the leading-edge vortices. The ground effect weakens the peak strength of the vortices due to incomplete development and causes them to move outwards (spanwise) while increasing their overall size. Additionally, the ground effect reduces the Strouhal number (St), indicating a slower vortex formation process. Furthermore, lift (CL) and drag (CD) coefficients increase as the wing descends towards the ground. The lift-to-drag ratio (CL/CD) also exhibits a rise with decreasing ground clearance (h/c), with this effect being more pronounced at lower angles of attack [19]. In a separate study, Tunse et al. [20] focused on the leading-edge vortex characteristics of underground influence. They observed early vortex collapse due to an intensified adverse pressure gradient on the suction side of the wing. The ground effect also increased turbulent kinetic energy at the wing surface, which is likely related to previous vortex collapse and complex flow structures. Finally, the average vertical velocity decreased with time as the wing approached the ground due to airflow blocking the gap between the ground and the underside of the wing.

Guerrero et al. [21] conducted a comprehensive study of the flow behavior around various realistic conventional road automobile designs, assessing the aerodynamic forces at play. Their study compared a baseline flat-underfloor configuration with three increasingly complex setups: a venturi diffuser with a moderate 7-degree angle, a combination of the venturi diffuser and diagonal side skirts, and finally, the same venturi diffuser integrated with frontal slot diffusers. The design featuring the venturi diffuser coupled with diagonal sealing skirts demonstrated the highest downforce coefficient, with a CL value of −0.887, showcasing a remarkable 1780 % improvement compared to the baseline model.

Therefore, the purpose of this study is to investigate the relationship between the diffuser and the rear tires numerically using CFD. The study also provides specific and quantitative results on the impact of different diffuser design parameters on drag and downforce. This will be presented using CFD to simulate airflow around a model of a Nissan Sunny or Versa car, modeled with high precision in designing software. The study includes a mechanism for improving the aerodynamic performance of this car, which is considered one of the most common cars in the Middle East, especially in the Gulf countries. Since the vehicle is designed in a way that gives modest aerodynamics and lacks stability at high speeds, a theoretical study was conducted on the mechanism of improving the aerodynamic properties using a double-element inverted wings diffuser, which is considered a novel point of this research, as the diffuser used is a multi-element wing with two inverted elements placed in different locations under the trunk of the car. End plates were also used to interact with the air coming from the wheels, direct the airflow from the car's underside to the back of the vehicle, and reduce eddies from the back wheels. Therefore, the findings showed that the ride height significantly affects the airflow through the diffuser, increasing base pressure and downforce production. The study also investigated the diffuser's effect on the car's stability, especially the rear side. The results showed that the diffuser significantly improved the car's stability, especially at high speeds. The remainder of this paper is organized as follows. Section 2 describes the Numerical Setup and methodology used in the study, including the numerical setup and method. Section 3 presents the results of the study. Section 4 discusses the results and their implications. Section 5 concludes the paper.

## 2 Methodology and numerical setup

**Fig. 1:**
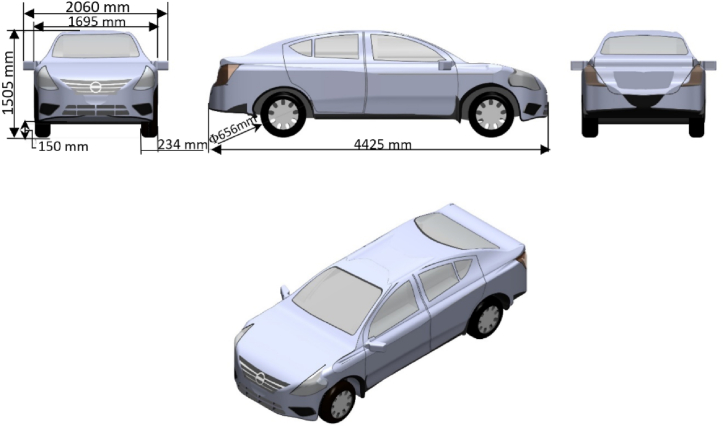
Baseline Nissan Sunny car.

The geometry of a current saloon car and a commercially available CFD code were used to identify how best to carry out the study as a numerical-only analysis. A realistic copy of the NISSAN Sunny vehicle was used as the geometry. An actual car has many complex elements underneath it, whereas the simulation model employed in this study was simpler. The main assumptions are listed in Table 1. See the model in Fig. 1.

**Table 1:** The assumptions made for numerical models.

Numerical Car Models	
Rotating wheels	Yes
Flat underbody surface	Yes
Side mirrors	Yes
Full-scale car size	Yes
The same material is used for all components of the car	Yes
The car's engine intakes air from the front of the vehicle	No
Exhaust gases are expelled behind the car.	No
The distance between the road and the car's chassis remains constant	Yes

**Fig. 2:**
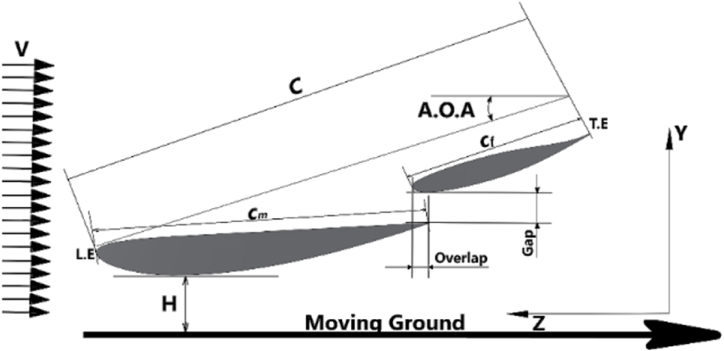
The double-element inverted airfoil used in the present study shows the definition of airfoil chord (c), ride height (h/c), angle of attack (α), and freestream velocity (V).

For the current study, the double-element inverted airfoil used is shown in Fig. 2. It is a highly-cambered inverted airfoil with two elements in a single slotted flap setup. The CLARK-Y smoothed (clarkysm-il) is the main element, and the profile type of LS (1)-0413 is the flap. The flap configuration has a finite trailing edge of 0.95 mm. The main element (c_m_) and flap (c_f_) have a chord length, the straight distance between the leading edge (nose) and trailing edge, of 300 mm and 165.7 mm, respectively. A total chord of 465 mm is used for all calculations for ease of calculation. Main element max thickness ratio11.7 % at 30.9 % of the chord. The second element of the wing has max. Thickness of 12.9 % at 37.5 % chord and a max. Camber of 2.4 % at 65 % chord. The angle of attack (α) or (A.O.A), the angle between the chord and the horizontal line, is positive for a nose-down rotation. Additionally, the accurate angles of attack for the main element (m) and the flap (f) will be +4° and +15.5° for a reference angle of attack (α) of 0° for the wing, respectively, based on the best lift-to-drag ratio, (Cl/Cd) for each element which these angles can provide [19]. The clearance between the ground surface and the lowest point on the suction surface of the aerofoil is defined as the ride height or ground-aerofoil clearance (h/c), with the aerofoil incidence set to zero degrees—flap location for the flap gap (δ_g_) and overlap (δ_o_). Furthermore, the wing extends 1350 mm in span and 2.89 aspect ratio (AR). In all scenarios, the free stream velocity was fixed at 30 m/s, resulting in a Reynolds number based on the vehicle length of about 1.3 × 10^7^.

**Fig. 3:**
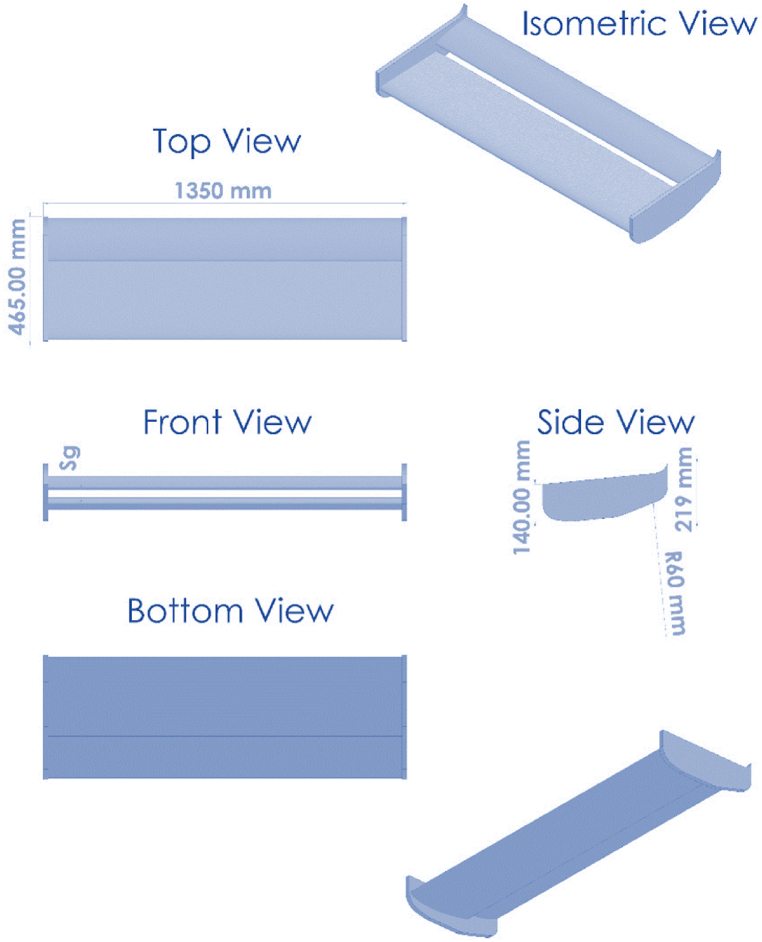
SOLIDWORKS model of the double-element inverted airfoil.

Fig. 2 displays a schematic of the under-body rear wing used in the current study. The mentioned wing model was created in SolidWorks 2022 and is represented with different views by the parameters in Fig. 3.

**Fig. 4:**
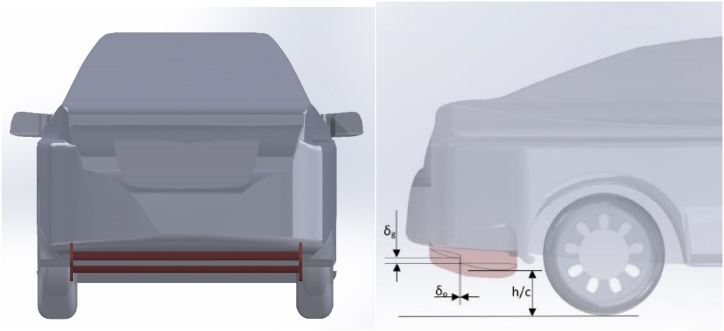
The double-element inverted wing on the Nissan Sunny model with parameters.

Fig. 4 shows the double-element employed on the Nissan Sunny model's rear under the trunk as an aerodynamic device to enhance aerodynamic performance. This device used different dimensions and riding heights to investigate the best aerodynamic design Table 2. Other sizes, such as height and width, were chosen based on the exterior design of this car. All of this device's dimensions and parameters are shown in Fig. 3, Fig. 4, where the double-element inverted wing is represented in red.

**Table 2:** Different cases of the double-element model.

Case	h mm	h/c	δo mm	δo/c	δg mm	δg/c
1	154	0.331	5	0.011	10	0.022
2	154	0.331	10	0.022	10	0.022
3	154	0.331	15	0.032	10	0.022
4	154	0.331	25	0.054	10	0.022
5	154	0.331	5	0.011	20	0.043
6	154	0.331	5	0.011	30	0.065
7	154	0.331	5	0.011	40	0.086
8	179	0.385	5	0.011	10	0.022
9	112	0.241	5	0.011	10	0.022
10	154	0.331	15	0.011	20	0.043
11	129	0.278	5	0.032	10	0.022
12	119	0.256	5	0.011	10	0.022

**Fig. 5:**
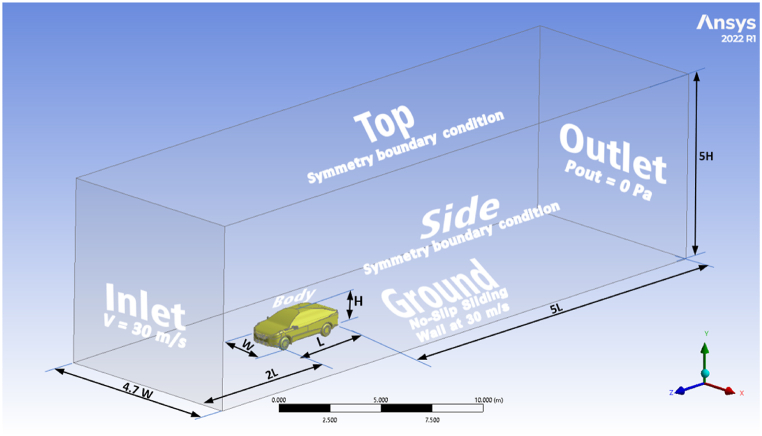
The computational domain of the NISSAN Sunny (Almera) is H = 1.505 m, W = 1.695 m, and L = 4.425 m.

The decision was made to create a wind tunnel simplified and idealized with a uniform rectangular cross-section for numerical simulations, aiming to be similar to the characteristics of the low-speed wind tunnel (LSWT) used for experimental validations [22]. The term "idealized" describes the wind tunnel domain's capacity to minimize solid blockage when introducing car geometry. Moreover, the domain's length ensures that the inlet and outlet remain adequately distant from the model, unaffected by the pressure field generated before the model, allowing undisturbed wake development. The blockage produced with the automotive geometry was around 4.25 % in the wind tunnel domain. The entire domain length was two vehicle lengths in front of the automobile and five vehicle lengths behind it, whereas the height is five times the car height, and the width is 4.7 times the car width, see Fig. 5 [23]. Additionally, the walls' symmetry conditions (the sides and top of the computational domain) prevent the development of wall boundary layers and keep the static pressure across the walls constant. A zero-shear slip wall condition was created. The ground boundary was given a no-slip moving wall boundary condition with a moving velocity of 30 m/s. The inlet velocity and the ground's translational speed were both the same. The wheels were also rotated at a rate that matched the inlet velocity. The downstream boundary was set with a gauge pressure of 0 Pa, and the diffuser car body was designated as a stationary wall with a no slip boundary condition.

**Fig. 6:**
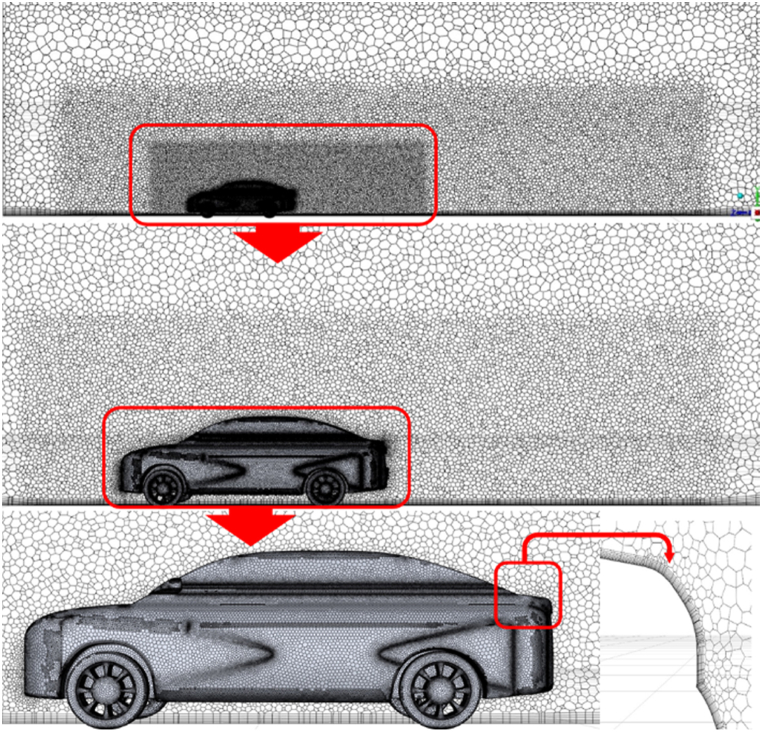
Polyhedral mesh with two virtual boxes and 15 inflation layers around the Nissan Sunny model and over the road (using first layer thickness).

**Fig. 7:**
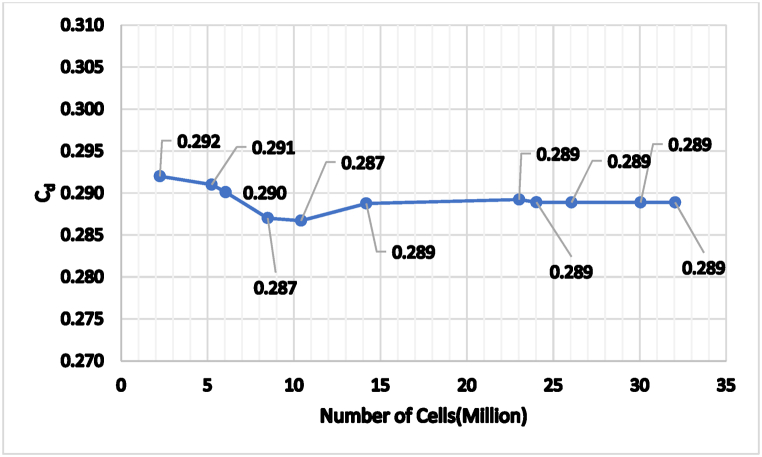
Grid Dependency for the Realizable k-ε Model.

The commercial ANSYS Fluent CFD meshing software built the domain's surface and volume mesh. As seen in Fig. 6, a hybrid grid comprising polyhedral and structured prism layers was utilized to construct the mesh. The largest mesh size was 0.001 m for the body, 0.0005 m for the airfoil, and 0.050 m away from the body (with a maximum of 0.020 m for the rest of the moving ground). A dynamic mesh was used to capture the moving ground. However, two "virtual boxes" were built around the model to increase the flow simulations' resolution. These "virtual boxes" had maximum mesh dimensions of 0.035 m and 0.075 m, respectively, the first one length of 12 m and width with a height of 2.5 m for each. The second box is 16 m long, 3 m wide, and has a height of 3.5 m. The mesh creation was carried out using ANSYS Meshing (version 2022 R1) at various levels of refinement. A y ^+^ value of 1 was used in the meshing approach, resulting in a first prism layer height of roughly 2.72e-5 m. The mesh quality was assessed using the aspect ratio, skewness, and orthogonality metrics. All cells have an aspect ratio less than 5, skewness less than 0.8, and orthogonality greater than 0.5. The simulated ride height affected the total grid size, the boundary layer prism layers number, and the exponential growth of these layers. The prism layers number and total cell count ranged from 15 to 30 and 12 million to 22 million, respectively, depending on the examined inverted wing ride heights (h/c = 0.256, 0.278, 0.331, and 0.385), with a growth rate ranging from 1.0 to 1.1. Sensitivity analysis was used to determine the ideal cell count. The realizable k-ε turbulence model is used in Fig. 7 to illustrate a grid dependency analysis for a typical computational domain for the maximum negative lift ride height (h/c = 0.331) [24].

A 2 % difference in drag coefficients was detected in this evaluation between the coarse mesh (about 12 million cells) and the fine mesh (roughly 18 million cells). Further refinement of the fine mesh to about 22 million cells resulted in a significantly smaller variation in drag coefficients (around 1 %) than the refined grids.

The car's geometry and half of the computational domain were utilized in all simulations to speed up the calculation procedure, with the ride height test cases using a minimum grid of about 12 million cells. Additionally, a sensitivity analysis on domain size was conducted. The downstream (outlet) border was adjusted at different distances: first at five times the car length (5L), then at seven times the car length (7L), and finally at nine times the car length (9L) [23]. The observed variation in drag coefficients among these domains was minimal, with a difference of less than 1 %. A smaller domain size was ultimately selected to optimize computational efficiency and reduce computing time.

## 3 Numerical method

**Fig. 8:**
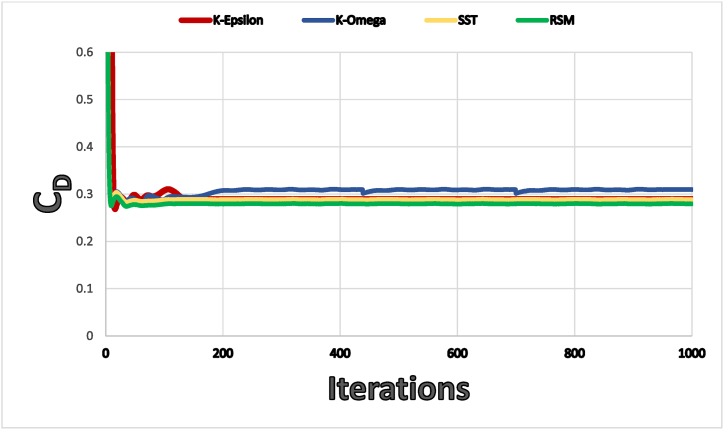
Drag coefficient for the baseline full scale model of the NISSAN sunny (Almera) using four types of turbulence model.

In this study, the solver used for the simulations and solving the average Reynolds average Navier Stokes equations (RANS) was Fluent 2022 R1 using the finite volume method. Four turbulence models were used: Realizable k–ε, standard k-ω, Shear Stress Transport k-ω (SST), and a Reynolds Stress Model (RSM). Previous researchers have widely applied these models to the aerodynamic behavior of cars, which would result in reasonable computational time. Fig. 8 shows the drag coefficient, CD, for NISSAN Sunny's full-scale baseline model using the four different turbulence models. After 200 iterations, there was minimal variation in the calculated drag coefficient. Fig. 8 also shows that all of the turbulence models (realizable k- ε, standard k- ω, SST k- ω, and RSM) used to simulate the NISSAN Sunny's baseline model offered good agreement with the officially published and experimental data, but the realizable k- ε result was the closest.

The realizable k- ε turbulence model is perfect for helpful study results in a respectable amount of time. Therefore, in the current study, the NISSAN Sunny (Versa) car modifications were all simulated using the k- ε turbulence model. The Realizable k-ε model was chosen because of its stability and quick convergence, as Cant and Pope [25] suggested. Realizable k-ε models have generated more accurate results than ordinary k-ε models in conditions involving severe streamline curvature, vortices, and rotating flows, such as the flow around the Nissan Sunny Car [26].

Second-order upwind discretization schemes were used to discretize the momentum, turbulent kinetic energy, and turbulent dissipation, and Fluent's coupled solver was utilized in the computations.

Convergence was reached when drag reached a stable level, and residuals stabilized at a sufficiently low level (below 10^−6^). Drag and lift are calculated as the average of the last 100 iterations.

To validate the CFD simulations, the drag coefficient of the baseline car was compared to the value published on the official Nissan Sunny (Versa) website [27]. The difference between the two values was less than 1 %, which indicates that the CFD simulations are accurate, see Table 3.

**Table 3:** Validation of results.

	Official NISSAN Sunny's Data	ANSYS Fluent Results			
		Realizable K-ε	Standard k-ω	SST k-ω	Reynolds Stress Model
**C**_**d**_	0.29	0.288	0.309	0.288	0.279
**Percentage Error (%)**		0.69	6.55	0.69	3.8

To further validate the CFD simulations, pressure coefficient (Cp) measurements were performed using a low-speed wind tunnel [22]. The wind tunnel experiments were conducted at an inlet velocity of 30 m/s (108 km/h), the same velocity used in the CFD simulations. The pressure distribution along the car's centerline was measured using a series of static pressure taps located along the centerline of the 1:18 scaled 3-D printed car model.

**Fig. 9:**
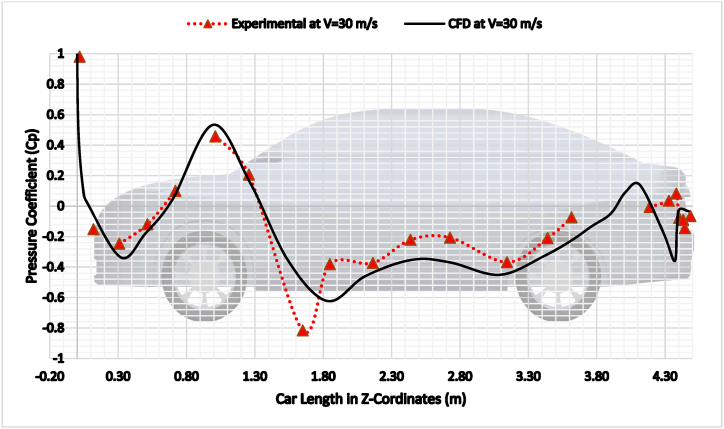
CFD Validation of car Model Center line with Wind Tunnel Experiments.

The measured pressure coefficients were compared to the simulated pressure coefficients and were in good agreement. The difference between the simulated and experimental pressure coefficients was less than 5%, see Fig. 9. This agreement is consistent with the agreement between the simulated and experimental drag coefficients.

## 4 Aerodynamic forces on vehicles

Bernoulli's equation states that the pressure of a fluid decreases as its velocity increases. This principle can be used to explain why wings generate lift.

The pressure coefficient (Cp) is a dimensionless parameter that can be applied to air moving at less than 0.3 times the speed of sound to study the flow of incompressible fluids. It is defined as follows:

(1) $$Cp=\frac{\left(p\;-\;p_{\infty }\right)}{\left(0.5\times \rho \times V^{2}\right)}$$

where *p* is the static pressure at a specific location within a flowing fluid to the pressure, *p*_*∞*_ is the static pressure in the freestream, *ρ* (rho) is the freestream air density, and *V* is the freestream velocity of the air.

Two major aerodynamic forces act on a car moving through the air: drag and lift. Drag is a force that opposes the car's motion, while lift is a force that acts perpendicular to the direction of motion. Drag and lift are influenced by the car's external design, frontal area, viscosity, air density, free stream velocity, and surface smoothness. The drag (CD) and lift (CL) coefficients are calculated as follows:

(2) $$CD=\frac{FD}{\left(0.5\times \rho \times V^{2}\times A_{p}\right)}$$

(3) $$CL=\frac{FL}{\left(0.5\times \rho \times V^{2}\times A_{p}\right)}$$

where *CD* is the dimensionless drag coefficient, *FD* is the drag force, ρ is the air density, *V* is the velocity of the oncoming flow, A_p_ is the projected area of the vehicle, *CL* is the dimensionless lift coefficient, and *FL* is the lift force.

These coefficients are important for designing aerodynamic cars. By understanding how these coefficients are calculated and how they affect a car's aerodynamic performance, engineers can design more efficient and effective vehicles.

## 5 Results

In the present study, the aerodynamic characteristics of a double-element airfoil installed under the car from the rear were analyzed, where the best dimensions between the wings, the overlap and gap distances, as well as the height of the wing from the ground, and the study of the effect of both the ground and wheels on the wing at fixed Reynolds number and angle of attack were based on previous studies [28] to obtain the best aerodynamic results and compare them with the baseline car model.

### 5.1 Drag and lift

**Fig. 10:**
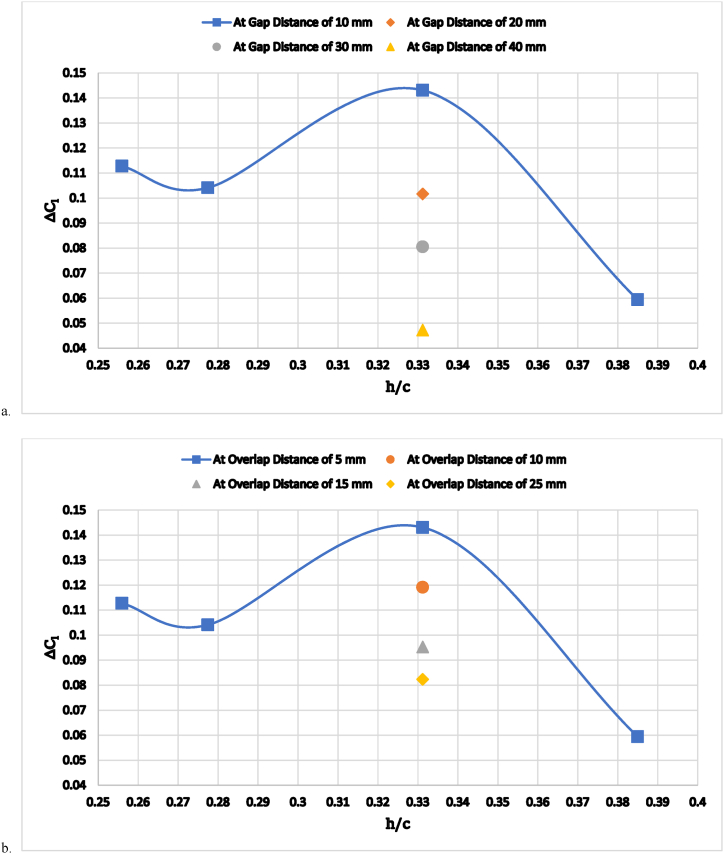
The increase in the lift coefficient, CL, as a function of wing ride height (a) at a constant overlap distance of 5 mm; (b) at a constant gap distance of 10 mm.

In Fig. 10 (a, b), Simulations were conducted for four cases for each ride height, each involving a different combination of gap distance and overlap distances. The results of each simulation were then compared, and the combination of gap distance and overlap distance that resulted in the highest change in lift or drag coefficient was chosen as the optimal combination.

For an overlap distance of 5 mm (Fig. 10 a) and a gap distance of 10 mm (Fig. 10 b), simulations were conducted for each ride height of 0.256, 0.278, 0.331, and 0.385. The maximum change in lift coefficient was at a ride height of 0.331, and this was considered the optimum case at constant overlap distances. In the cases of overlaps 10 mm, 15 mm, and 25 mm, only simulations with a ride height of 0.331 % were conducted.

**Fig. 11:**
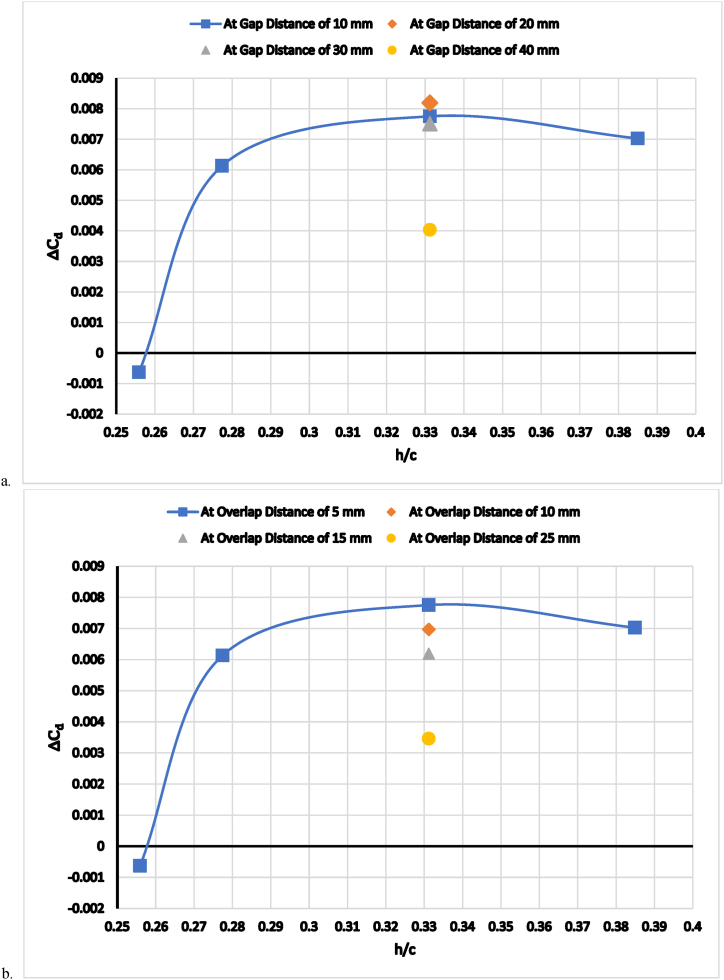
The increase in the drag coefficient as a function of wing ride height (a) at a constant overlap distance of 5 mm(b) at a constant gap distance of 10 mm.

Fig. 10a and b show the increment in negative lift coefficient, ΔCl, over the baseline car model without a diffuser wing case. As can be seen, downforce increases when the double wing diffuser gap and overlap distances, δg and δo, are reduced while the ride height, h/c, distances remain constant at 0.331. The flap and main element angle of attack, α, were fixed at 15.5° and 4°, respectively, because these angles provide the best lift-to-drag ratio [29]. Fig. 11 (a, b) shows the effect of flap-to-main element overlap (a) and gap (b) distances on the car's drag. The optimum overlap and gap distances were discovered to be 5 mm and 10 mm, respectively. It is, therefore, interesting to note that the slope of the increase in negative lift increases at a diffuser overlap distance of less than 5 mm and that the slope of the increment of drag decreases beyond this distance, but the dimensions of the car prevent taking small overlap distances, as shown in Fig. 11b.

**Fig. 12:**
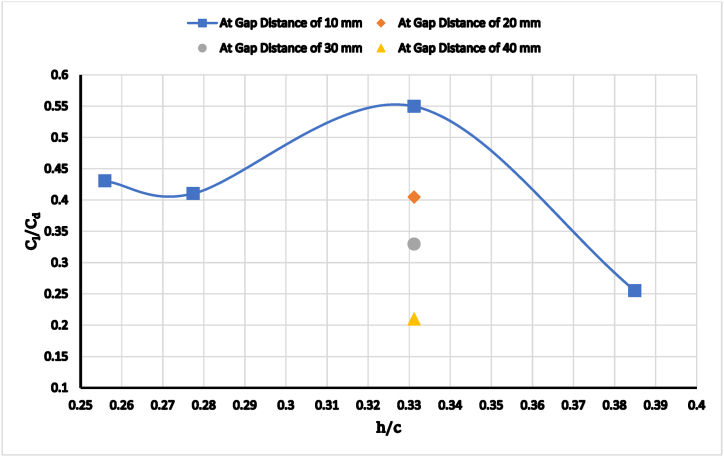
The lift-to-drag ratio versus wing ride height at a constant overlap distance.

Fig. 12 shows the lift-to-drag ratio; efficiency improves with ride height until a certain point, after which it begins to decrease. Regarding gap distance, the most aerodynamic configuration is at 10 mm, with lift-to-drag dropping off at other distances. As the gap distance increases or decreases, the lift-to-drag ratio decreases due to the formation of separated zones or multiple vortices that increase drag and decrease lift. At 0.33118 (h = 154 mm), the optimum wing ride height to the ground, the lift-to-drag ratio is reduced by creating enclosed zones that increase drag as the wing height increases at specific points close to the car body.

In general, the diffuser wing reduces drag by about 2.7 %. It increases downforce by ten times at the best double-element design, with overlap distance and gap distance of 5 mm and 10 mm, respectively, at a wing height of 154 mm. These results are significant in comparison to Christoffersen et al. results [13], who found a maximum lift change of 0.025 and a drag change of 0.005. This means that the double-element diffuser wing developed in this study is much more effective at improving downforce and reducing drag than the previously considered single-element diffuser Christoffersen et al., [13].

Another novel finding of this study is that the lift-to-drag ratio is improved with ride height until a certain point, which begins to decrease. This suggests an optimal ride height for the diffuser wing, which depends on the specific design of the diffuser and the car.

### 5.2 Wake development

**Fig. 13:**
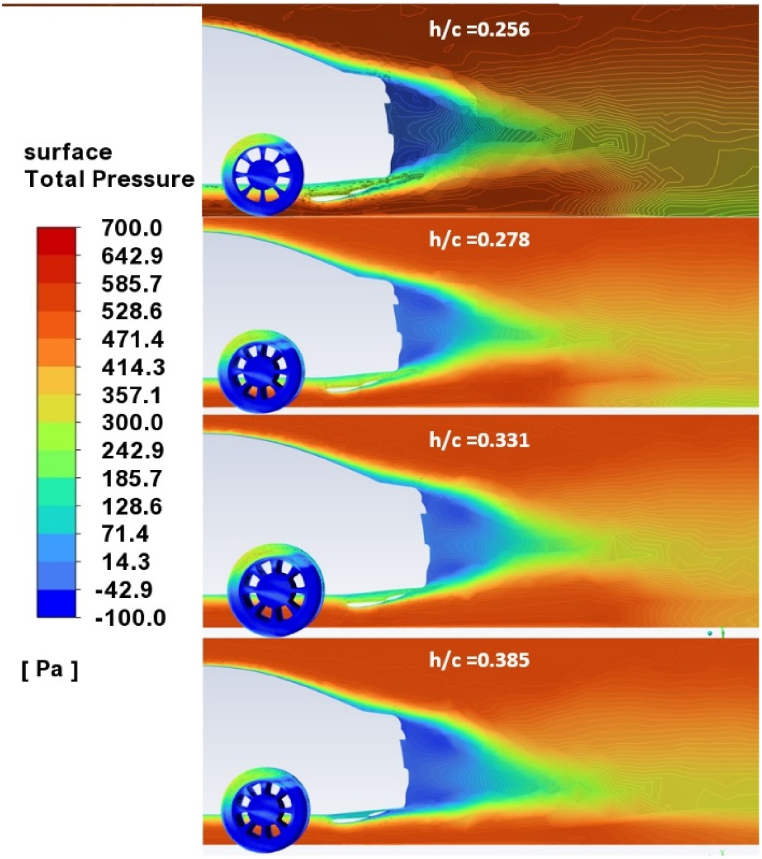
Total pressure contours in the car center plane.

**Fig. 14:**
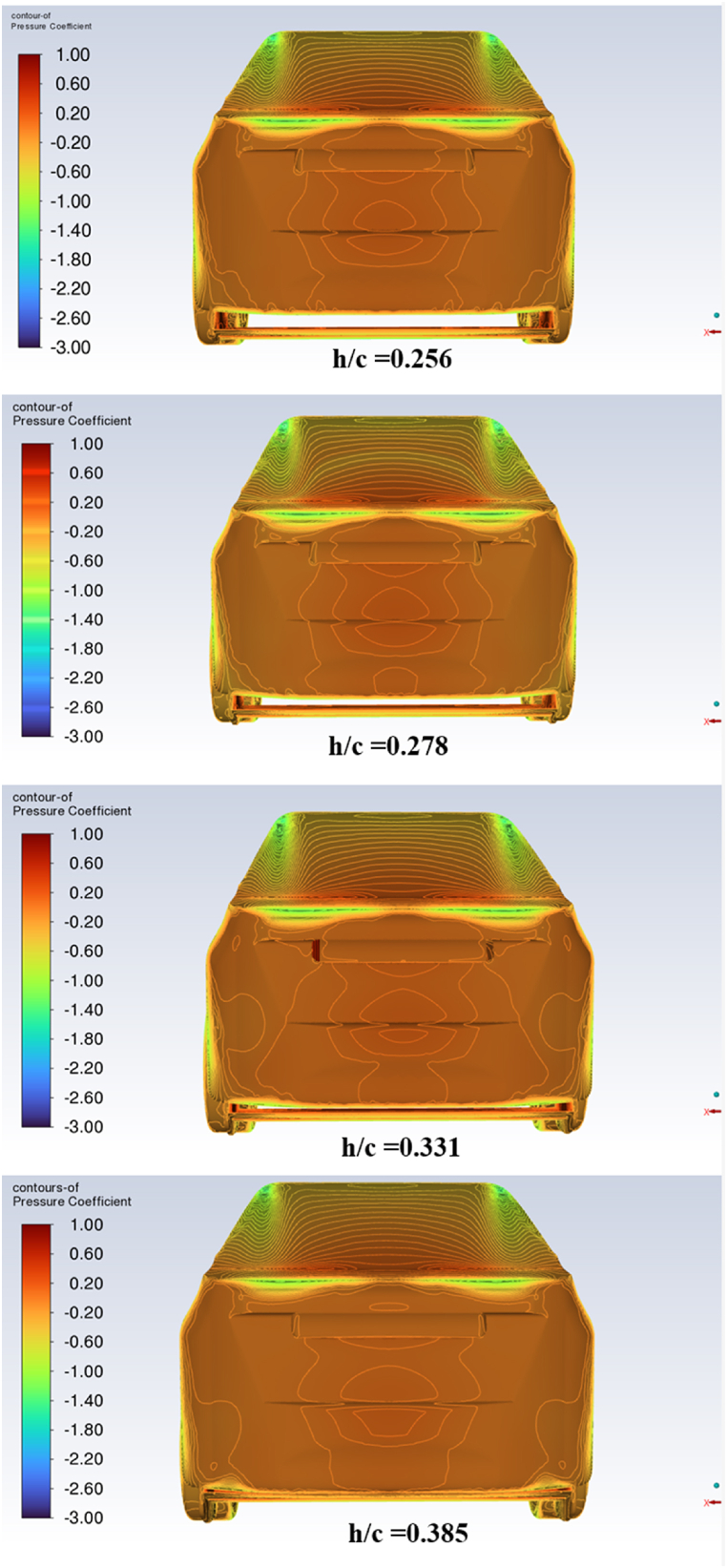
The pressure coefficient at the car base.

Fig. 13 shows the contours of total pressure in the wake's center plane for wing diffuser ride height per cord length (h/c) values of 0.256, 0.278, 0.331, and 0.385, respectively. The results show that lowering the diffuser ride height reduces the wake size left by the car because it increases the pressure under it. This increased pressure helps to push the airflow closer to the ground, which reduces the size of the separation bubble that forms behind the car. This separation bubble is what causes the wake, so reducing its size reduces the size of the wake. As seen in Fig. 14, this has the result of lowering base pressure. The figure shows the vehicle's base pressure coefficient, CP, at four different diffuser ride heights: 0.256, 0.278, 0.331, and 0.385, respectively. This leads to an increase in aerodynamic drag, as seen in Fig. 11. The diffuser ride heights are 0.256, 0.278, 0.331, and 0.385 from top to bottom. At the max ride height of 179 mm, the wing diffuser almost comes into contact with the car's trunk, increasing the blue region's size and causing rising pressure drag. The wake development results of this study show that lowering the diffuser ride height reduces the size of the wake left by the car. This is a novel result, as previous studies have not investigated the effect of diffuser ride height on wake development. The results also show that lowering the diffuser ride height lowers base pressure. This is also a novel result, as previous studies have not investigated the effect of diffuser ride height on base pressure. The combination of these two results suggests that lowering the diffuser ride height can improve the aerodynamic performance of cars by reducing wake size and lowering base pressure.

Regarding the comparison with existing literature, results align with Christoffersen et al. [13] in terms of diffuser angle impact. They showed reduced base pressure and shorter wake behind the car with increasing angle. This strengthens our conclusion that diffuser geometry significantly influences wake development and base pressure.

### 5.3 Trace of surface pressure

**Fig. 15:**
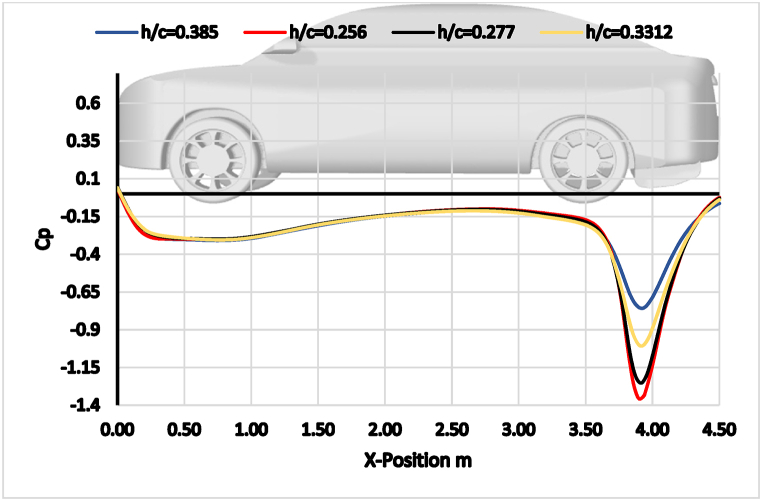
The static pressure coefficient along the underbody and wing diffuser surface.

The static CP distribution along the underbody and diffuser surfaces of the car is illustrated in Fig. 15. The graph presents the CP for each studied diffuser ride height (0.256, 0.277, 0.3312, and 0.385) along the centerline of the rear diffuser wing. The highest suction pressure peak at the diffuser beginning is observed for the diffuser ride height of 0.256 (h = 119 mm), as confirmed by the lift coefficients shown in Fig. 10.

Furthermore, it is found that decreasing the gap and overlap distances of the diffuser's double element leads to an increase in downforce. This is a novel result, as it suggests that the interaction between the surface pressure and underbody can be used to improve the diffuser's performance. As the wing ride height decreases, the negative peak of the CP also grows. As a result, at this airfoil height, it is believed that the wing settings may have only a minor impact on the diffuser's performance, provided the wing is not stalling.

The main difference between this study's results and Christoffersen et al. [13] is the magnitude of the pressure coefficient. The results of this study show a higher peak CP at the start of the diffuser. This is likely due to the different diffuser geometries used in the two studies. The diffuser used in this study diffuser has a smaller gap and overlap distance between the double elements, which leads to a stronger interaction between the surface pressure and underbody.

### 5.4 The effect of rear tires wakes

**Fig. 16:**
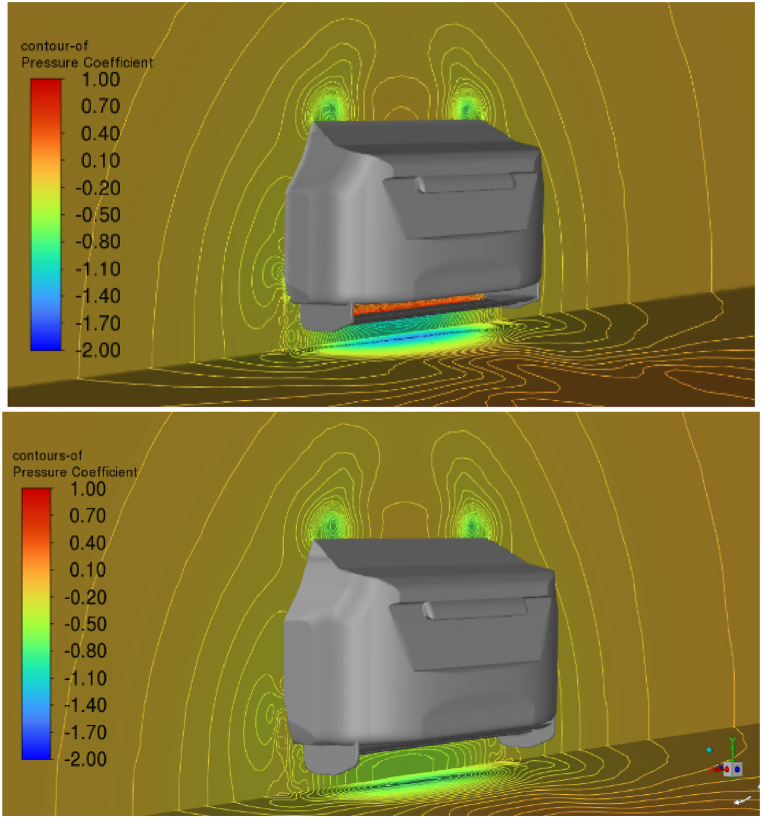
The total pressure coefficient at the beginning of the diffuser.

Fig. 16 shows that the rear wheel wakes significantly affect the diffuser performance. In the figure, a plane displays the total pressure coefficient just below the car's rear wheels. The rear tire wakes to appear to be pulled towards the body of the car because of the decreased pressure produced at lower diffuser ride height. The diffuser performs poorly because of the lower flow rate caused by the flow's reduced momentum.

However, as shown in Fig. 10, the concentration of tire wake appears to have little or no impact on the lift reduction. A possible reason for this phenomenon is that when diffuser ride heights expand, a reduction in midline static pressure along the underbody, as illustrated in Fig. 15, covers the increase in regions affected by the rear tire wakes. In the case of drag, however, a greater rear tire wake and the corresponding drop in total pressure led to a higher drag coefficient. This explains the noted rise in drag at reduced diffuser ride heights, as shown in Fig. 11.

### 5.5 Contour plots

**Fig. 17:**
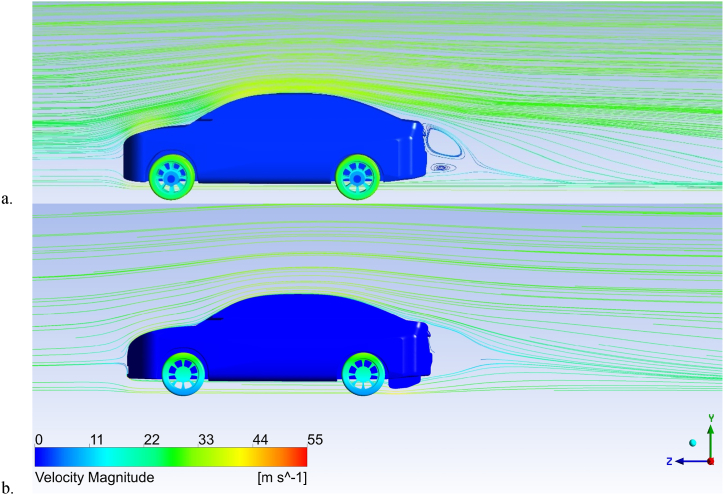
Streamline around the nissan sunny (Versa) for (a) baseline model, (b) rear diffuser.

Fig. 17 (a, b) shows the streamlines around the Nissan Sunny (Versa) baseline model (a) and the modified model with a double-element diffuser (b). The simulations were conducted with an inlet air velocity of 30 m/s. The baseline model generates two spanwise vortices behind the vehicle due to a low pressure zone. The diffuser modification produces a more streamlined wake by filling some wake regions and the low pressure area. The greater pressure behind the rear bumper causes the air coming from the diffuser outlet to go downward, which reduces airflow over the vehicle. The diffuser modification achieves a more streamlined effect than the baseline model, resulting in lower air resistance.

**Fig. 18:**
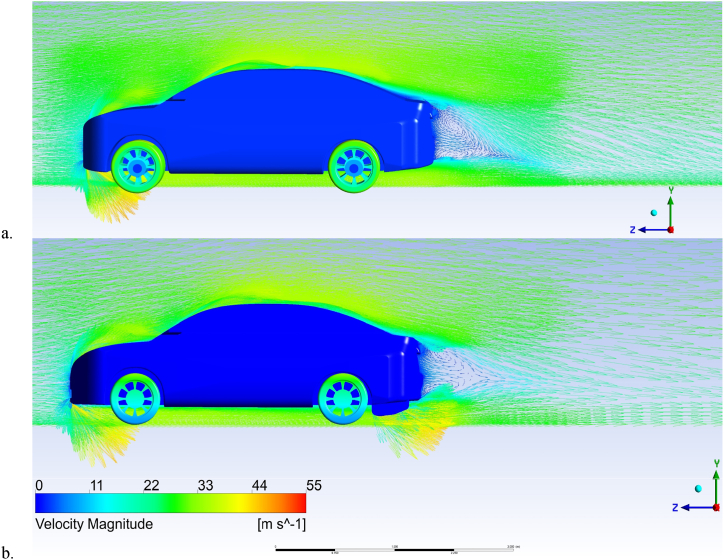
Velocity Vector Around the Nissan Sunny for (a) Baseline model, (b) Rear Diffuser.

Fig. 18 (a, b) shows the velocity field around the Nissan Sunny (Versa) baseline model (a) and the modified model with a diffuser (b), evaluated on the model symmetry plane for each case study. The diffuser modification produces a more homogenous velocity distribution behind the saloon than the baseline model.

## 6 Conclusion and future outlook

The study revealed a significant influence of the diffuser wing's vertical position on airflow. Overlap and gap distances between the wing elements critically affected drag, negative lift, base pressure, and downforce production. The optimal configuration, achieved at a 154 mm ride height, 10 mm gap distance, and 5 mm overlap, resulted in a remarkable ten times the downforce increase and 2.7 % drag reduction. Lower ride heights triggered a noticeable shift of the rear tire wake towards the car center, potentially affecting performance. A longitudinal fence could address this issue. Notably, the diffuser modification yielded a more streamlined wake, filling low pressure regions and achieving lower air resistance than the baseline model. This highlights the potential of this design for improved car aerodynamics, offering both performance and fuel efficiency benefits.

These findings highlight the novelty of the double-element diffuser wing design. Its effectiveness in improving downforce and reducing drag surpasses previous single-element designs. Additionally, the findings on the effect of diffuser ride height on wake development and base pressure are new and could be used to improve the aerodynamic performance of cars.

One concrete potential application of the results is in the design of race cars. Race cars must have high downforce and low drag to be competitive. The double-element diffuser wing could improve the aerodynamic performance of race cars by increasing downforce and reducing drag. Another potential application of these results is in the design of fuel-efficient cars. Fuel-efficient cars need to have low drag to maximize fuel economy. The double-element diffuser wing could be used to reduce fuel-efficient cars' drag, improving their fuel economy.

Beyond the current study, exciting avenues for further research beckon. Investigating diverse diffuser designs' impact on ground effect and overall aerodynamic performance remains crucial. Exploring active flow control strategies holds the potential to unlock even greater diffuser effectiveness. Evaluating real-world performance under varied driving conditions, road surfaces, and speeds is essential for practical implementation. Integrating the diffuser into the Nissan Sunny's underbody and addressing manufacturing, installation, and maintenance challenges will inform feasibility. Comparing the diffuser's performance to other passive flow control techniques like vortex generators or strakes offers valuable insights. Exploring applicability to other vehicle types like trucks, buses, and motorcycles broadens the potential impact. Conducting a life cycle assessment will evaluate the environmental footprint of the diffuser. Investigating its economic feasibility for production vehicles ensures responsible development. Future work could focus on developing self-cleaning mechanisms beyond the initial concept, exploring options like automated brush systems, anti-fouling coatings, or aerodynamic designs that naturally shed debris. Collaborating with safety experts to develop advanced control algorithms that account for the diffuser's influence on vehicle dynamics will ensure safe and predictable behavior across diverse driving scenarios. Finally, developing noise-dampening technologies specifically for the diffuser to mitigate acoustic emissions without compromising efficiency, potentially involving soundproofing materials or targeted acoustic suppression systems, will be crucial for widespread adoption. By pursuing these diverse research avenues, we can unlock the full potential of this innovative diffuser design, paving the way for significant advancements in car aerodynamics with benefits for both performance and fuel efficiency.

## Funding statement

This study receives no external funding for this research.

## Data availability statement

The data associated with this study has not been deposited into a publicly available repository. The datasets used and/or analyzed during the current study are available from the corresponding author upon request.

## CRediT authorship contribution statement

**Mustafa Sabeeh Abood:** Writing – review & editing, Writing – original draft, Validation, Software, Resources, Methodology, Investigation, Data curation. **IhsanYahya Hussain:** Supervision.

## Declaration of generative AI and AI-assisted technologies in the writing process

While preparing this work, the authors used Google Bard to improve language and readability. After using this tool/service, the authors reviewed and edited the content as needed and took full responsibility for the publication's content.

**Table:** Nomenclature

*CD*	Drag Coefficient	*CFD*	Computational Fluid Dynamics
*CL*	Downforce, negative lift coefficient.	*h*	Height, mm
*V*	Inlet Free stream velocity. m/s	*Re*	Reynolds Number
*P*	The free stream pressure, Pa	*δg*	Gab distance, mm
*c*	The wing chord length, mm	δo	Overlap distance, mm
*h/c*	Ride height	*α*	angle of attack, degree
*V*	jet velocity, m/s	*L*	Car Length, mm
MRF	Moving Reference Frame	*Y+*	Dimensionless wall distance parameter
k	Turbulent kinetic energy	*ε*	Turbulent dissipation rate
AR	Aspect ratio	*CP*	Coefficient of Pressure
FL	Lift force, N	*FD*	Drag force, N
(RANS)	Reynolds Average Navier Stokes equations	*ρ*	Air density, kg/m^3^

## Declaration of competing interest

The authors declare the following financial interests/personal relationships, which may be considered as potential competing interests Mustafa sabeeh abood reports administrative support and writing assistance were provided by the University of Baghdad, Iraq.
